# Towards an Understanding of the Low Bioavailability of Quercetin: A Study of Its Interaction with Intestinal Lipids

**DOI:** 10.3390/nu9020111

**Published:** 2017-02-05

**Authors:** Gillian T. Rich, Maria Buchweitz, Mark S. Winterbone, Paul A. Kroon, Peter J. Wilde

**Affiliations:** 1 Institute of Food Research, Norwich Research Park, Norwich NR4 7UA, UK; gillian.rich@ifr.ac.uk (G.T.R.); mark.winterbone@ifr.ac.uk (M.S.W.); paul.kroon@ifr.ac.uk (P.A.K.); 2 Universität Stuttgart, Analytical Food Chemistry, Allmandring 5B, 70569 Stuttgart, Germany; maria.buchweitz@lc.uni-stuttgart.de

**Keywords:** mixed micelles, UV-visible spectra, pyrene fluorescence, small intestine, bioaccessibility, phosphatidylcholine, lysophosphatidylcholine, bile salts

## Abstract

We have studied the uptake of quercetin aglycone into CaCo-2/TC7 cells in the presence and absence of mixed micelles that are present in the human small intestine. The micelles inhibited the transport of quercetin into the cells. To gain an understanding of why this is the case we examined the solubilisation of quercetin in micelles of differing composition and into pure lipid phases. We did this by using the environmental sensitivity of quercetin’s UV-visible absorption spectra and measurement of free quercetin by filtration of the micellar solutions. The nature of the micelles was also studied by pyrene fluorescence. We found that the partitioning of quercetin into simple bile salt micelles was low and for mixed micelles was inhibited by increasing the bile salt concentration. The affinity of quercetin decreased in the order egg phosphatidylcholine (PC) = lysoPC > mixed micelles > bile salts. These results, together with the innate properties of quercetin, contribute to an understanding of the low bioavailability of quercetin.

## 1. Introduction

Quercetin is a flavonoid that is found in all plant foods. As with other flavonoids, its role in higher plants is to function as an antioxidant [1]. In humans it is postulated that not only is it an antioxidant, but it can also act directly on cells as an anti-inflammatory agent [2]. It has properties demonstrated in vivo and in vitro consistent with protection against cardiovascular disease [3]. There is also evidence for anticancer [4] and antiviral [5] effects. However, the bioavailability of quercetin is low [6] and its absorption can be affected by macronutrients [7]. In plants, quercetin is in the form of glycosides, which are converted to the aglycone by β-glycosidases in the intestine before being absorbed into the enterocytes [8,9]. Here they are metabolised to quercetin conjugates. Previously, we studied quercetin aglycone solubilisation in simple bile salt (BS) micelles of composition relevant to the duodenal lumen [10]. In the present work aspects of quercetin’s solubilisation are extended to mixed micelles, PC liposomes and lysoPC micelles, which are all lipid phases found in the small intestine at different stages of digestion. Since flavonoids are absorbed and conjugated by human epithelial intestinal CaCo-2 cells [11,12], we studied quercetin uptake into CaCo-2/TC7 cells in the presence and absence of mixed micelles with the aim of getting information about whether micelle solubilisation influences its bioavailability. To our knowledge, this is the first study of the effect of lipid micelles on quercetin uptake by intestinal cells.

**Figure 1:**
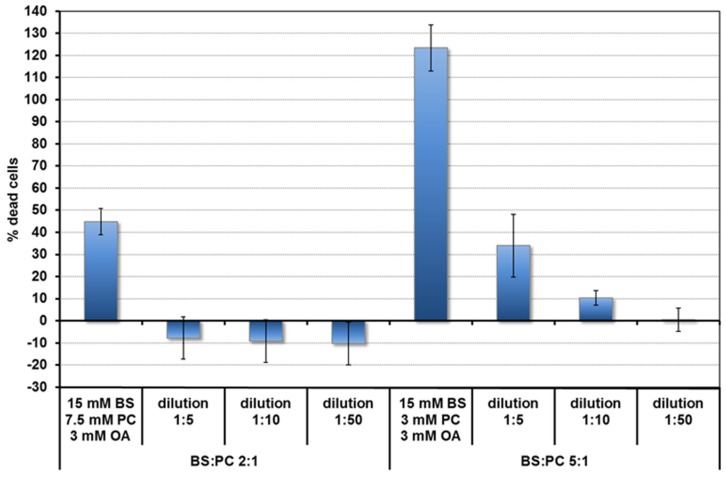
Toxicity of mixed micelles (fed state) to Caco-2/TC7 cells depending on BS:PC ratio and concentration.

The UV-visible spectrum of quercetin is sensitive to the pH and polarity of its environment [10]. The structure of quercetin and its spectra as a function of pH and presence of BS are shown in Figures 1 and 3A of our previous publication [10]. The molecule is planar, consisting of two aromatic rings (A and B) linked by a γ-pyrone ring. We have found that the wavelength of maximum absorption of the long wavelength peak (Peak B) has a bathochromic shift when quercetin moves from an aqueous phase into a more hydrophobic phase (for example bile salt micelles). Thus, we were able to show that the adsorption site on simple bile salt micelles of composition mimicking the hydrophobicity of bile salts in humans was less polar than that on sodium dodecyl sulphate micelles. (This was confirmed by pyrene fluorescence, where the vibronic fine structure of the spectra depends on the polarity of the pyrene environment.) The effect of changing pH is most clearly seen in the short wavelength peak (Peak A). The first ionisation of quercetin (HQ ➔ H^+^ + Q^−^) gives rise to a peak at 270 nm. Therefore, the ratio of the maximum absorbance of peak A to that at 270 nm gives a measure of the relative amounts of HQ and Q^−^. Using this ratio, we can measure the relative affinity of the ionised and neutral forms of quercetin for micelles or other lipid phases and from peak B assess the relative polarity of the binding sites.

In vivo, quercetin’s bioavailability is enhanced if fed with oils [13,14,15]. Its oil/water partition coefficient favours partition into oil (log P = 1.8), but this value is amongst the lowest of those for flavonoids [16]. This reflects quercetin’s relatively polar nature. It is possible that the role of the oil is to stimulate bile production so as to solubilise the quercetin in lipid micelles. Alternatively, in spite of the rather low partition coefficient, the oil and in particular the oil–aqueous phase interface could provide a quercetin store for subsequent passage into the enterocytes. Indeed, we [10] and others [17,18] have shown that in simple micelles and lipid membranes quercetin is solubilised close to the polar groups of the organised structures. This may also be true of other nutrients and drugs that have an intermediate polarity between strongly hydrophobic molecules (such as vitamin D and cyclosporin) and hydrophilic water soluble molecules (such as vitamin C and aspirin). Therefore, quercetin is not only of interest as a flavonoid, but also as a model compound for other similar compounds of interest. When considering the relative bioavailability of compounds, their positive affinity for lipid phases found in the small intestine is often referred to as solubilisation. However, the interaction may be a binding process, as is the case for quercetin and pyrene. This could be thought of as a solubilisation in the region of the binding sites. Therefore, in this paper we have used the terms solubilisation and binding to mean the same: a positive interaction with the lipid phases.

## 2. Materials and Methods

### 2.1. Materials

Quercetin (anhydrous), sodium oleate (OA), bile salts (sodium taurocholate (NaTC) dried to constant weight over calcium oxide and sodium glycodeoxcholate (NaGDOC)), pyrene (99.9%), ascorbic acid and dimethyl suphoxide (DMSO) were from Sigma-Aldrich (Gillingham, UK). DMSO, quercetin and OA were stored under argon. Phosphatidylcholine (PC) made from egg lecithin (grade 1) and lysophosphatidylcholine (lysoPC) made from egg lecithin were from Lipid Products (South Nutfield, UK). Dulbecco’s phosphate buffered saline (DPBS), Ca^2+^ and Mg^2+^ free, (10×) were from Gibco, Life Technologies Corp (Paisley, UK). The LDH assay was from Sigma-Aldrich. Other chemicals were of analytical or HPLC grade. Centrifugal filter units (30 K molecular weight cut-off, size 2 and 15 mL) were supplied by Merck Millipore (Darmstadt, Germany).

### 2.2. Methods

#### 2.2.1. Preparation of Solutions

As previously described [10], special precautions are needed in preparing solutions containing quercetin: exclusion of oxygen and restricted exposure to light. Thus oxidation is minimised [19]. Solutions and solvents were deoxygenated by sonication and kept under argon in amber glassware. Quercetin was dissolved in DMSO and diluted to 45 or 22.5 µM into aqueous solutions with stirring so the DMSO concentration was 0.1%.

Micellar solutions were prepared by evaporating under nitrogen solutions of PC or lysoPC (in chloroform/methanol) ± OA (in methanol), followed by overnight evaporation in a vacuum chamber that had been purged three times with nitrogen. For mixed micelles a bile salt solution in DPBS at the required pH was then added to the dried lipids and the mixture incubated at 37 °C for 20 to 60 min on a shaker rotating at 170 rpm with a few glass beads. The bile salt solution contained NaTC and NaGDOC in the molar ratio of 1:1.12. As described previously, this composition mimicked the average hydrophobicity of bile salts in human bile [10]. Liposomes made of PC alone and PC with OA were sonicated under nitrogen and (equilibrated with DPBS) to translucence or transparency using a Status 200 ultrasonic homogeniser. After equilibration of the micelles the quercetin solution was added and the solutions equilibrated at 37 °C. Equilibration was faster after sonication for 30 s in a bath sonicator (Sonicor Instrument Corporation, Copiague, NY, USA). For cell culture and centrifugal filtration experiments, 100 and 200 µM ascorbic acid, respectively was included in the solutions. The size of liposomes was measured by dynamic light scattering using a Malvern Nano-ZS Zeta Sizer (Malvern Instruments, Malvern, UK).

#### 2.2.2. Solubility Measurements

The solubility of quercetin at 37 °C was assessed by incubating solid quercetin in media containing 200 µM ascorbic acid for up to 24 h with shaking and at intervals taking samples, which were centrifuged at 10,000× *g*. Using measured extinction coefficients, solubilities were calculated from the UV-visible spectra of the supernatants, which were filtered through syringe PDVF filters (0.2 µm pore size). Quercetin adsorbs to the filters, but after two filtrations the quercetin concentration in the filtrates was constant. The method had the advantage of rapidity and showing whether the quercetin had suffered damage (e.g., oxidation) during the incubation.

#### 2.2.3. UV-Visible and Fluorescence Spectroscopy

Spectral measurements were made at 37 ± 1 °C as described previously [10] using either a Uvicon xs or Perkin Elmer, Shelton, CT, USA Lambda 25 spectrophotometer for UV-visible spectra and a Perkin Elmer LS55 luminescence spectrophotometer for pyrene fluorescence. The excitation wavelength for pyrene fluorescence was 310 nm. The fine structure of pyrene’s emission spectra between 370 and 400 nm is dependent on the local environment, such that the ratio of the maxima of peaks III (around 384 nm) and I (around 373 nm) are sensitive to the polarity of the environment surrounding the pyrene molecule [20]. The peak height ratio of peak III /peak I (*F_R_*) in the pyrene fluorescence spectra were measured to calculate the relative polarity of the pyrene binding site in the micelles [20]. Low values (<1) indicate a polar environment (Water *F_R_* = 0.62) and higher values (>1) indicate a less polar environment (Isopropyl alcohol *F_R_* = 1.1; *n*-Hexane *F_R_* = 1.67) [20]. Data were corrected for scattering effects by measuring spectra against blanks containing no quercetin or pyrene for UV-visible spectra and fluorescence, respectively.

#### 2.2.4. CaCo-2/TC7 Cell Culture and Quercetin Uptake

Cells were grown on a six well plate at 37 °C in supplemented Dulbecco medium (1% non-essential amino acids, 1% l-glutamine, 100 IU/mL penicillin, 100 µg/mL streptomycin and 10% (*v*/*v*) foetal calf serum) under an atmosphere of 5% CO_2_, as described previously [11]. Cells were used after 21 days for quercetin uptake experiments. Due to stability issues of quercetin in DMEM^+^ media, uptake was done in DPBS at a pH appropriate to mimicking the proximal duodenum (pH 7.15).

Treatment solutions were prepared by adding 100 mM ascorbic acid and 45 µM quercetin to the micellar solutions followed by careful mixing for 1 min after each addition. Cells were washed with DPBS and 2 mL treatment solution was added to each well. The control for each plate had freshly prepared treatment solution, but without the micelles. After incubation at 37 °C for 30 min, the treatment solutions were removed and the cells washed with DPBS twice. Cells were harvested in 0.4 mL water and transferred to amber Eppendorf tubes, containing 75 µL methanol:acetic acid (1:2) covered with argon. The cell samples were mixed thoroughly for one minute, sonicated at room temperature, mixed again and centrifuged at 15 °C, 13,000 rpm for 10 min. Potential instability of quercetin in solution was avoided by analysing the samples immediately by HPLC and the samples were kept at 15 °C (sampler temperature). (15 °C was found to be the optimum temperature for storage of quercetin in the methanol–acetic acid–water mixtures.)

Toxicity of the micelles was assessed by the LDH assay. Based on the toxicity results (Figure 1) and literature [21,22], concentrations of lipids in the duodenum for the fed and fasted states, bile salt and lipid concentrations were chosen for the quercetin uptake experiments. These are shown in Table 1. They are 10 times lower than the concentrations expected in undiluted duodenal contents. The BS and PC concentrations are based on the averages found after fasting (FA state) and after a meal (fed, FE state) (summarised in [21]). Food, particularly fat, stimulates bile production. The BS duodenal concentration in the FE state is around 15 mM. The BS: phospholipid ratio is between 2:1 and 5:1. In the FA state concentrations are 10 times lower. Fatty acids come mainly from fats in the food. Since there is some fat (e.g., triglyceride) hydrolysis in the stomach [22], OA was also added for the FE state at 0.3 mM. This value was estimated from the amount of gastric hydrolysis (9%–24%) [22] in a meal containing 18 mmol of fat releasing 3.2–8.6 mmol long chain fatty acid. It underestimates the effect of dilution in the intestines, but this is compensated for by further fatty acid release from fat digestion in the duodenum.

#### 2.2.5. Centrifugal Ultrafiltration of Micelles and Liposomes

Inter-micellar/inter-liposomal concentrations of quercetin in systems containing mixed micelles, lysoPC alone or PC alone were determined by the centrifugal ultrafiltration method of Donovan et al. [23] using 30KD cut off membranes. The centrifugation was carried out at 37 ± 1 °C at 3000 rpm (1600 g). Quercetin binds to the filters. Therefore, it was necessary to do successive filtrations (3–5) with fresh filtrant until the filter had adsorbed quercetin to equilibrium. Thereafter, the quercetin concentration in the filtrates reached a constant value. This process could be shortened by pre-equilibrating the filters overnight with the quercetin solution to be filtered. In order to avoid significant changes in concentration of the lipids and quercetin, the filtrate volume for each filtration was restricted to ≤10% of the initial volume of the filtrant.

#### 2.2.6. Quantification of Quercetin and BS

BSs and lipids were stable in stored solutions. This was not the case for quercetin in spite of storing material anaerobically and in the dark. Freezing tended to give irreversible quercetin precipitation. Therefore, it was important to assay quercetin as soon as possible. Quercetin in Caco-2/TC7 cells was measured by HPLC-DAD (method 1). For the filtration experiments quercetin was quantified by UV-vis spectroscopy using the maximum absorbance of Peak B for filtration with the 15 mL centrifugal filters and by HPLC (method 2) with the 2 mL filters.

HPLC Method 1: Quercetin analyses were performed with an Agilent 1100 series HPLC system equipped with a DAD. The compounds were separated on a Phenominex Luna C18 (2) 100 A column (250 × 4.6 mm ID, 5 µm; Phenominex, Torrance, CA, USA) with a C18 security guard (4 × 3 mm ID, Phenomenex) at a constant temperature of 30 °C and flow rate of 1 mL/min. Solvent A was 50 mM ammonium acetate buffer, adjusted to pH 5.1 with acetic acid, and solvent B consisted of 2% THF and 0.1% acetic acid in acetonitrile (*v*/*v*; LiChrosolv, Merck, Darmstadt, Germany). The initial solvent composition was 17% B for 2 min. Separation was achieved using a gradient from 17% B to 25% in 5 min and further to 35% and 50% in 8 min and 5 min, respectively. B was increased to 100% in 5 min and kept constant for 5 min before re-equilibration to initial conditions in 20 min. The injection volume was 100µL and quercetin was monitored at 370 nm.

HPLC Method 2: To simultaneously quantify bile salts and quercetin the isocratic method of Rossi et al [24] was used with a Grace Alltima HP C18 (250 × 4.6 mm, 5 µm, 25 cm; C & P) column without a pre-column. The eluent consisted of 10 mM sodium phosphate buffer and methanol 25:75 (*v*/*v*) at pH 5.35. At a column temperature of 30 °C and a flow of 0.7 mL/min, compounds were separated within 20 min. Injection volume for the FE (1.5 mM BS with PC and OA, 45 µM Q) and the FA state (0.3 mM BS with PC and OA, 45 µM Q) were 15 µL and 20 µL, respectively. The retention times of TC and GDOC were determined at 200 and 210 nm at about 8.4 min and 17 min respectively. Quercetin was detected at 370 nm, exhibiting a retention time of about 5.0 min. Individual compounds were quantified using calibration curves of TC, GDOC and quercetin. The column was cleaned successively with 100% water, 25% acetonitrile, 50% acetonitrile and 75% acetonitrile.

## 3. Results

### 3.1. Properties of Quercetin Relevant to Its Bioavailability

The *pK_a1_* of quercetin at physiological conditions of ionic strength and temperature is 7.08 [10]. In the absence of solubilising agents, the solubility of quercetin in aqueous solutions is low and pH dependent. Adding 1 mM BSs (a concentration below the cmc of the BS mixture) increases the solubility, particularly at a lower pH. Solubility in food oils is higher (see Table 2).

### 3.2. Uptake of Quercetin into Cells

**Figure 2:**
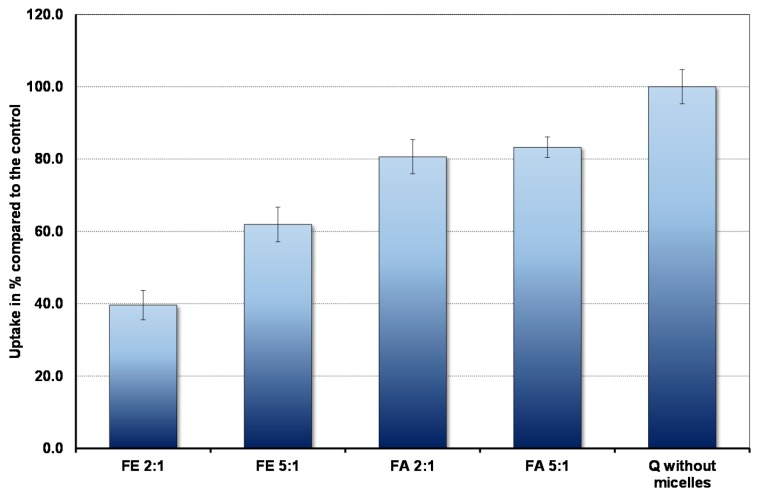
Pyrene fluorescence in fed state mixed micelles with BS: PC ratio of (**A**) 2:1 and (**B**) 5:1. The OA concentrations relative to the BS concentrations are as in Table 1.

The results are shown in Figure 2. In the context of bioavailability it is interesting that the cells absorb markedly less quercetin in the fed state, when duodenal micelle concentrations are high, compared to the fasted state. Micelles inhibit absorption so that even under fasting conditions uptake is less than that observed in the complete absence of micelles. Furthermore, the ratio between PC and BS plays an important role. The higher the proportion of PC, the lower the uptake.

### 3.3. Nature of the Micelles and Quercetin–Micelle Interactions

#### 3.3.1. Pyrene Fluorescence

**Figure 3:**
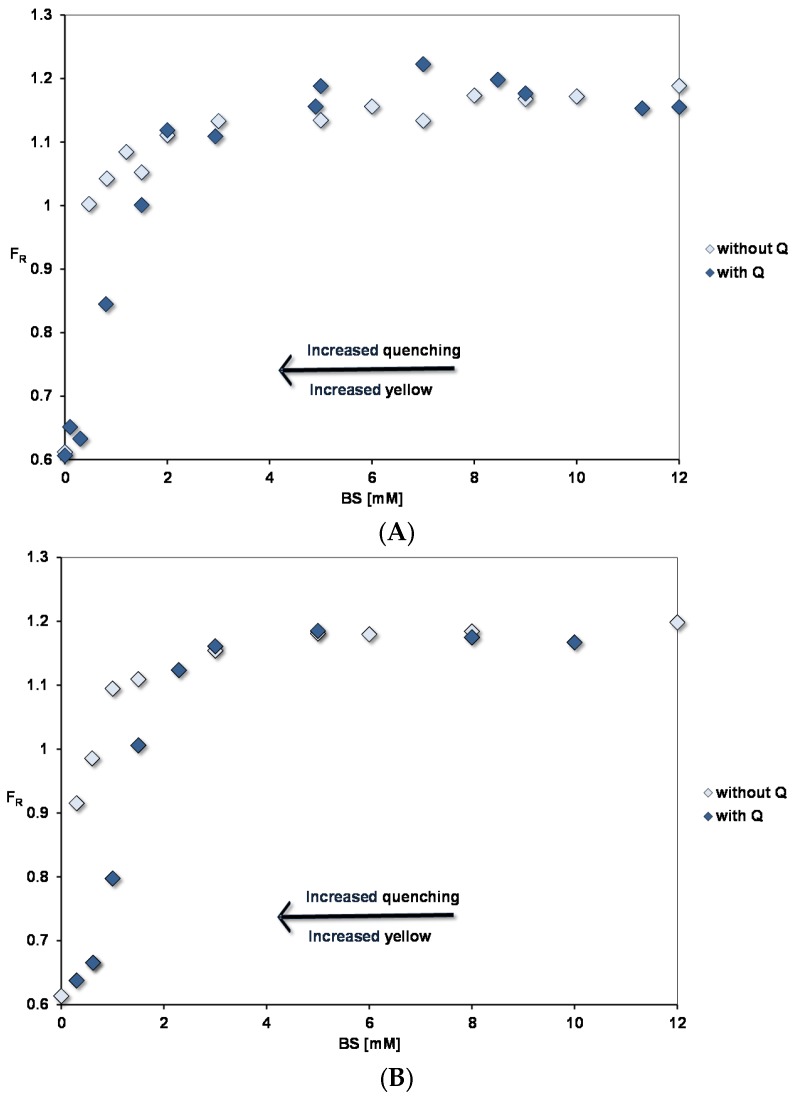
The amount of quercetin absorbed in 30 min by Caco-2 cells in the presence and absence of micelles (control).

Figure 3 shows the pyrene fluorescence intensity ratio, *F_R_* (Peak III/ Peak I) as a function of micellar concentrations corresponding to the fed (FE) state at pH 7.15. Concentrated micellar solutions were diluted to the required concentrations and the micelles contained BSs, PC and OA in the ratios defined in Table 1. The spectra show that a binding site for pyrene starts to form at extremely low micelle concentrations and in this region there is no significant difference between FE 2:1 and FE 5:1 conditions. We interpret this as the lipid mixtures having a very low critical micelle concentration (cmc). Constant *F_R_* values are reached at > 3 mM BS with FE 5:1 micelles showing a slightly higher *F_R_* than FE 2:1 (1.17 for 5:1 and 1.14 for 2:1).

Adding 45 µM quercetin reduces F_R_ in the low concentration region. This is associated with quenching of the pyrene fluorescence and an increased yellow colour of the micellar solutions, which fades at higher concentrations where there is no significant quenching. As described earlier [10], interaction of quercetin with negatively charged micelles will favour binding of the uncharged quercetin (HQ). This results in a yellow colour as the acid–base equilibrium is disturbed in the aqueous phase towards charged quercetin (Q^−^), which absorbs at 270 and 380 nm. In simple micelles, quercetin-pyrene interactions show the opposite trends: enhanced quenching and yellow colouration as the BS micelle concentration is increased.

Pyrene fluorescence was also studied for PC liposomes and lysoPC micelles, which indicated at the maximum *F_R_*, the polarity of the lipid phases increased in the order BS < FE5:1 micelles < FE2:1 micelles < PC liposomes < lysoPC micelles (see Table 3).

#### 3.3.2. Quercetin Absorption Spectra

**Figure 4:**
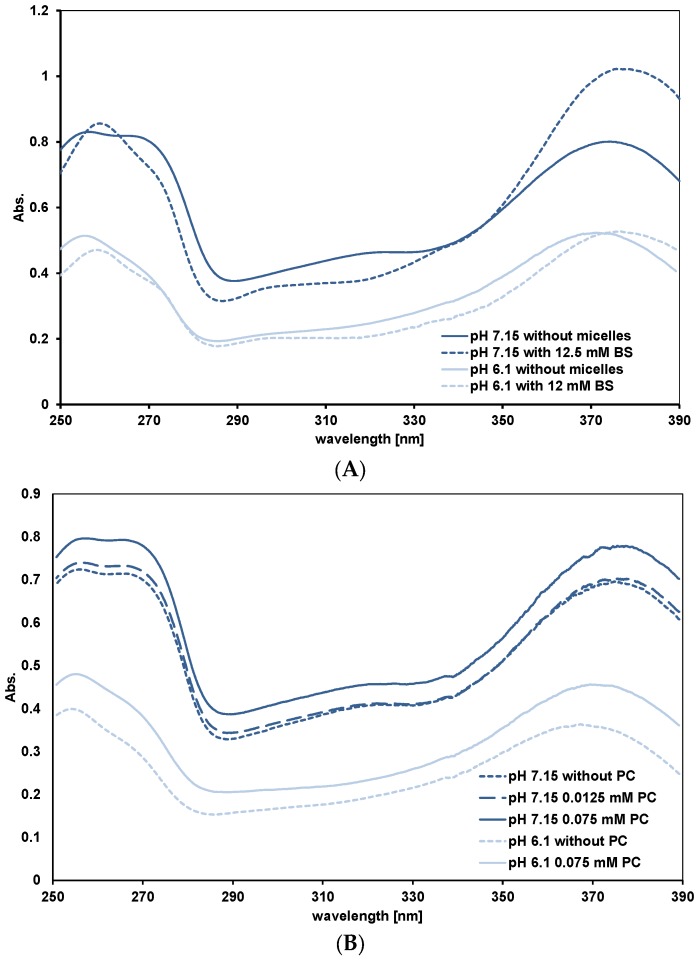
UV-visible spectra of quercetin in micellar solutions FE 2:1 (**A**) and PC liposomes (**B**). In (**A**) the PC and OA concentrations relative to the BS concentrations are as in Table 1.

*Peak A for assessing the Q^−^/HQ binding to lipid phases*. As explained in the Introduction, the shape of peak A gives information about the relative affinity of HQ and Q^−^ for the binding sites. For all mixed micelles we observed the same pattern. This is shown in Figure 4A for FE 2:1 micelles. At pH 7.15, as the mixed micelle concentrations are increased, the absorbance at 270 nm decreases. This is what we observed with simple BS micelles and means HQ is preferentially bound to the micelles. At pH 6.1, where there is little Q^−^, the 270 nm absorbance does not change significantly with micelle concentration. Quercetin in PC liposomes at pH 7.15 behaves differently. As the PC concentration is increased, the peak at 270 nm does not become a shoulder and the 270 nm absorbance, relative to the maximum absorbance of peak A does not change (see Figure 4B). This means Q^−^ and HQ are binding to the liposomes. Adding OA to PC, in the ratios 5:1 and 2:1 (PC:OA) changes the spectra to that characteristic of preferential HQ binding (results not shown). At pH 6.1, as for the micelles, peak A reflects only HQ binding to the liposomes. Simple micelles of lysoPC behave as mixed micelles. This is unexpected as PC and lysoPC have the same head groups where quercetin binds. The reason for this is mentioned in the Discussion.

**Figure 5:**
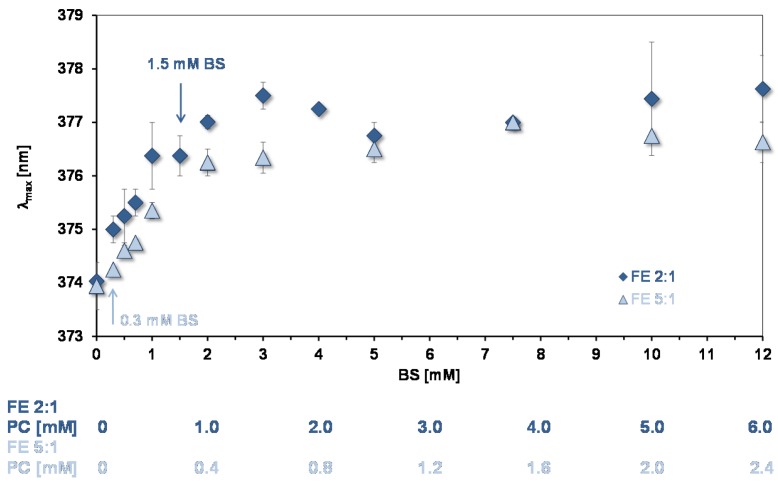
Bathochromic shift of peak B as a function of mixed micelle concentration (pH = 7.15). The arrows show the concentration of micelles used in the experiments of quercetin uptake by CaCo-2/TC7 cells. The OA concentrations relative to the BS concentrations are as in Table 1.

*Peak B for assessing the environments of binding sites for quercetin and aggregation state of lipids in mixed micelles.* The wavelength of maximum absorption of quercetin (λ_max_) of peak B gives information on the environment of quercetin bound to lipids and can be used to assess their state of aggregation [10]. Figure 5 shows data from experiments showing how λ_max_ changes as a function of the concentration of mixed micelles. As for simple micelles [10] there is a bathochromic shift as the lipid concentration increases showing that quercetin is partitioning into the mixed micelles. At BS concentrations >3 mM a plateau is reached, the same concentration that pyrene reported for reaching a plateau. Therefore, at >3 mM the environment of the absorption sites for the two probes are not changing with increase in the number of micelles.

As for pyrene, the increase in λ_max_ shows that micelles start to form at low lipid concentrations. However, the initial slopes in Figure 3 and Figure 5 indicate that quercetin has a much lower affinity for the micelles than pyrene does. The same situation was found for the simple BS micelles [10].

The mixed micelle λ_max_ plateau values for Peak B are summarised in Table 4 (column 3). The values are not significantly different, implying that in spite of the different lipid composition the hydrophobicity of the quercetin binding sites are similar.

**Figure 6:**
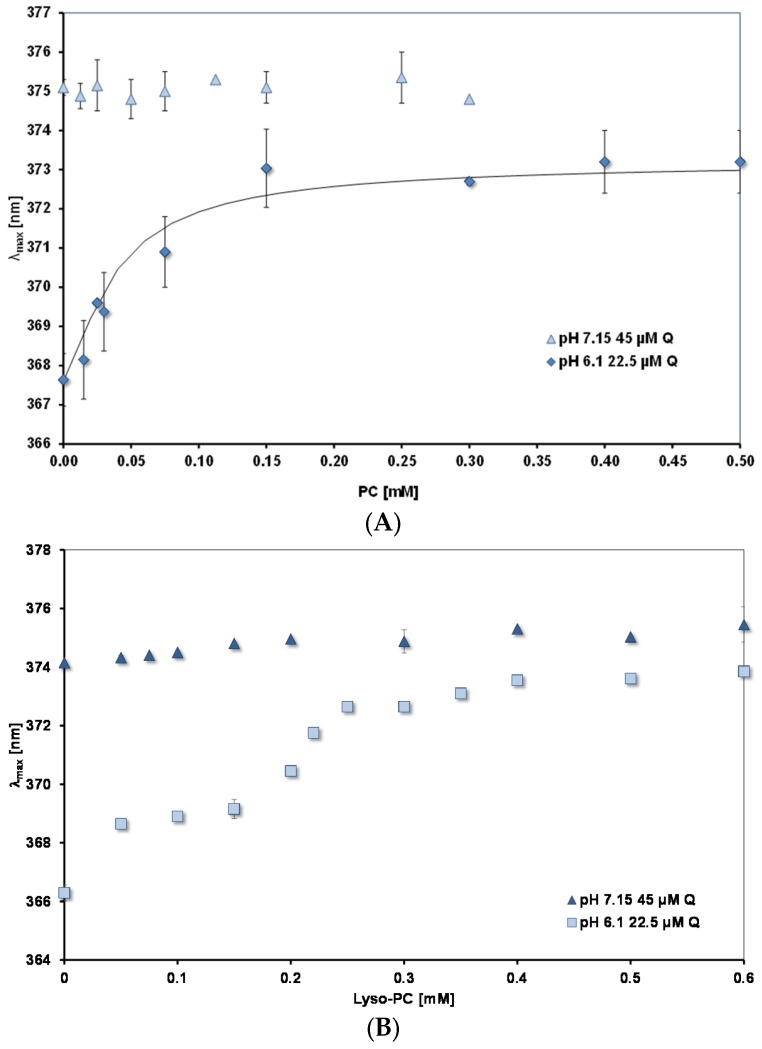
λ_max_ for peak B as a function of lipid concentration. (**A**) PC; (**B**) Lyso-PC. The continuous black line in (**A**) is calculated from a binding constant of 45 mM^−1^.

*PC and lysoPC interaction with quercetin.* Figure 6A,B show how λ_max_ changes as the concentrations of PC and lysoPC, respectively are increased. Measurements were done at two pH values: 7.15 and 6.1. For both lipids the plateau levels of λ_max_ are reached at much lower concentrations than for mixed micelles. (Note the different scales of the concentration axes in Figure 5 and Figure 6). For lysoPC at pH 6.1, where uncharged quercetin (HQ) predominates, there is evidence that the environment of the quercetin binding site undergoes a transition to a more hydrophobic nature between 0.3 and 0.4 mM. Pyrene fluorescence shows similar changes (spectral results not shown, but see Table 3 for an increase in F_R_ between 0.3 and 0.5 mM).

At the higher pH of 7.15, where there is 54% charged quercetin, Q^−^, as the lipid concentration is increased there is no significant change in λ_max_ from the value measured in the absence of lipid (374–375 nm). In both cases the plateau values for λ_max_ are significantly lower than those measured for the mixed micelles (see Table 4).

### 3.4. Affinity of Quercetin for Micelles and PC

Table 4 (column 4) shows the filtration results giving the amounts of quercetin bound to mixed micelles, PC liposomes and lysoPC micelles. The results were independent of the volumes filtered (100–500 µL). (This was not true for the BS concentrations in the filtrates, where their concentration increased with filtration volume. We conclude that for dilute model bile solutions the filtration method is not suitable for measuring intermicellar BS concentrations.)

We have found that at the lipid concentrations used, the presence of oleate did not affect quercetin binding to the micelles (results not shown). As expected, fewer micelles in the fasted state give less bound quercetin than in the fed state. For both the fed and fasted states, increasing BSs in the micelles inhibits quercetin binding. Replacing some of the PC with lysoPC causes an increase in binding. However, the change is not statistically significant. The quercetin binding results indicate that the affinity of quercetin for the lipid structures decreases in the order: PC = LysoPC > mixed micelles > BS.

For PC liposomes, where the lipid surface has a constant composition, the binding of quercetin at equilibrium can be described by the equation, *K*_b_ = (Q PC)/((Q)_free_.(PC)_free_), where *K*_b_ is the binding constant; (Q PC) and (PC)_free_ are the concentration of PC sites occupied and unoccupied, respectively, by quercetin; (Q)_free_ is the concentration of free quercetin in the aqueous phase. If we assume the bathochromic shift is proportional to the concentration of bound quercetin, (Q PC) = k (Δλ/Δλ_max_) and at the plateau all the quercetin is bound, then the data in Figure 6A at pH 6.1 can be fitted to give a value for *K*_b_. The continuous curve in the figure shows the best fit, giving *K_b_* = 45 mM^−1^.

## 4. Discussion

We have studied the binding of quercetin to lipid phases present in the small intestine and found there is preferential binding of uncharged quercetin (HQ) over ionised quercetin (Q^−^) to mixed and lysoPC micelles. The same is true for simple BS micelles [10]. Only in the case of PC liposomes do we find evidence for Q^−^ binding. A consensus view is that quercetin binds to the surfaces of organised lipid phases [10,17,18,25]. Only in the case of planar lipid membranes, where there is solvent present is there evidence for HQ penetrating between acyl chains [26]. The hydrophobicity of the binding sites decreases in the order BS > mixed micelles > PC = lysoPC and the affinity of quercetin for the lipid phases follows the reverse order. This is understandable because quercetin is a relatively polar molecule and will have an affinity for more polar surfaces. The bile salt mixture we have used mimics the average hydrophobicity of bile salts found in human bile. Quercetin has a low affinity for these BS micelles. However, the solubility data (Table 2) and our previous paper [10] indicate monomeric BSs can interact with uncharged quercetin (HQ).

Our approximate estimate for the affinity of unionised quercetin, HQ, for PC liposomes, expressed as a binding constant, is relatively high (45 mM^−1^). It is calculated from the best fit to the data over the whole range of concentration of PC studied and assumes all the quercetin is bound at saturation. If about half the PC is available for quercetin binding, the saturation of sites at about 0.15 mM PC by 22.5 µM quercetin suggests (75/22.5 = 3.3) PC molecules form each binding site. Half the PC molecules being available assumes uni-lamellar liposomes. The fact that transparent and translucent liposome suspensions give the same results is evidence that this is true. (The size of the transparent liposomes was 150 nm; translucent liposomes were not measured.)

Spectra at pH 7.15 (Figure 4B) indicate that ionised quercetin, Q^−^, adsorbs to the liposomes. This contrasts with mixed micelles, where there is no evidence of Q^−^ binding. The reason for the difference lies in the negative charge carried by the conjugated bile salts. The disc model [27] for the mixed micelles consisting of phospholipid, fatty acid and bile salts postulates a lipid bilayer structure with the potentially exposed hydrophobic fatty acid chains coated with bile salts. Quercetin preferentially binds to the surface of the bilayer part of the disc as it has a low affinity for the bile salts and the negative charge on the bile salts precludes Q^−^ binding. In fact, our attempts using UV-visible spectroscopy to measure a *K*_b_ for HQ binding to simple BS micelles gave inconsistent results because the affinity was so low (Using the fluorescent properties of quercetin which are more sensitive than UV-visible spectra would be a better method to measure *K*_b_ for quercetin—simple BS micelle interactions).

The importance of negative charge influencing quercetin adsorption is shown by OA addition to PC liposomes giving an inhibition of Q^−^ binding. The observation that OA does not significantly affect quercetin adsorption to mixed micelles is presumably due to the negative charge on the bile salts. Simple micelles of lysoPC showing preferential binding of HQ is unexpected, given that lysoPC and PC have the same polar head groups. LysoPC is susceptible to hydrolysis and it may be that some long-chain fatty acid was present in the micelles.

When mixed micelles are diluted, the intermicellar concentration of BSs is reduced. This means the micelle size increases as the lipid lamellae have fewer available BSs to shield the hydrophobic fatty acid chains from the aqueous phase. At low concentrations, as micelles are forming from phospholipid and bile salts, phospholipid lamellar polar surface will predominate and form a substrate for quercetin and pyrene binding. This explains the increased quenching of pyrene by quercetin as they both adsorb to the surface, increasing the chance of their mutual interaction. (Whether this is by fluorescent energy transfer or exciplex formation is yet to be determined.) At higher concentrations there is less lamellar surface for adsorption of quercetin and pyrene. The situation is different for simple bile salts, where the bile salt concentration must be high enough to form a surface for adsorption. This is why quercetin quenching of pyrene fluorescence for simple BS micelles shows the opposite trend to mixed micelles—increased quenching at high micelle concentrations.

Our results shed light on why the bioavailability of quercetin is low. To be bioavailable, a nutrient must first be bioaccessible (that is, solubilised) so it can be absorbed by the enterocytes. As a nutrient passes down the gastrointestinal tract it will be subjected to changing conditions of pH and solubilising agents (micelles and lipids). In this context, and in the light of our results on quercetin’s properties and solubilisation, we discuss here how quercetin’s bioaccessibility changes as it passes from the stomach to the distal small intestine and how the presence of oil can enhance its bioavailability. We suggested in the Introduction that the enhancement of quercetin bioavailability by oil might be due to the oil stimulating bile secretion into the duodenum. Our results indicate that this hypothesis is false. Bile components inhibit quercetin uptake into CaCo-2 cells.

*In the stomach* the pH is less than 4. With a *pK_a1_* of 7.08, quercetin will be unionised (HQ) and free HQ can pass through the stomach wall. However, HQ is poorly soluble so it will tend to precipitate. Nevertheless, quercetin absorption from rat stomach has been observed [28]. Quercetin’s solubility in oil is higher. Therefore, in oil it is protected from precipitation. Lipolysis by gastric lipase is limited [29] so quercetin in oil can pass through to the duodenum.

*In the proximal duodenum* lipid bilayers and micelles (simple and mixed) are present [30]—all potential solubilising structures. However, the pH is >7 so quercetin will ionise with Q^−^ ≥ HQ. Q^−^ could potentially reach the enterocytes in the free state or bound to PC, but diffusion through the negatively charged mucosal membranes will be restricted. We have found that mixed micelles, mimicking those in the upper small intestine, inhibit uptake of quercetin into CaCo-2 cells, which are considered to be able to model small intestine enterocytes [31,32]. Therefore, it seems even mixed micelles do not provide a pathway for enhancing quercetin absorption. We have shown that monomeric bile salts interact with unionized quercetin. The negative charge of the bile salts will further inhibit the diffusion of quercetin into the enterocytes. The lipolysis of emulsified oil by pancreatic lipases is known to enhance the passage of oil-soluble nutrients to micelles [33]. Therefore, although the micelle solubilisation of quercetin is low, oil can provide a quercetin reservoir for slow release of HQ, to overcome the limitations we have described.

*Moving towards the distal small intestine*, the pH first falls and then becomes alkaline. In the acid region, diffusion of HQ into the enterocytes is possible, but inhibited while there are still significant concentrations of micelles and monomeric bile salts, as described above. Increasing concentrations of lysolipids can potentially solubilise quercetin, but they must compete with the tendency of quercetin to precipitate. The reservoir of oil is diminished by lipolysis so provides a smaller source of protected quercetin. At the terminal ileum, where the pH is >7.4 [34], the quercetin will be more soluble but will not pass easily across the mucosal membranes.

Studies on quercetin uptake in different regions of the rat small intestine from fats loaded with quercetin have shown that most quercetin is absorbed in the ileum [14,35]. As the uptake media were the same for each section of the intestine, this effect was attributed to the smaller thickness of the mucus layer in the ileum. It would be of interest to measure uptake from media with the different concentrations of lipids appropriate for the different intestinal regions.

Of course, there are other factors that should be taken into account to understand quercetin’s bioavailability. For example, the environment of quercetin in the intestines is altered by the presence of foods. As well as fats, proteins and carbohydrates can alter the kinetics and absorption of plant phenols [7]. Further, we have not considered the possibilities of endocytosis of insoluble quercetin across the mucosal membranes and the effect of cholesterol, which is present in bile. The colon provides another site for absorption, but efficient metabolism of quercetin by the enterobacteria [36] will limit the amount that can be absorbed.

## 5. Conclusions

In conditions pertaining to the duodenum, mixed micelles inhibit the uptake of quercetin into CaCo-2/TC-7 cells. The affinity of quercetin for lipid phases found in the small intestine decreases in the order PC = LysoPC > mixed micelles > simple BS micelles. It is uncharged quercetin that is preferentially bound to the micelles. Taking into account the affinity of quercetin for different lipid phases and its innate properties, we can understand the inhibition of quercetin uptake by the cells and extrapolate to conditions pertaining to the whole gastrointestinal tract to explain its low bioavailability and how it is enhanced by the presence of oil.

## Figures and Tables

**Table 1:** Lipid concentrations and BS/PC ratios used for quercetin uptake into Caco-2/TC7 cells.

	BS:PC	(OA) mM	(PC) mM	(BS) mM
Fed state (FE)	2:1	0.3	0.75	1.5
Fed state (FE)	5:1	0.3	0.3	1.5
Fasted state (FA)	2:1	0.03	0.15	0.3
Fasted state (FA)	5:1	0.03	0.06	0.3

BS, bile salts; PC, phosphatidylcholine; OA, sodium oleate.

**Table 2:** Solubility of quercetin under different conditions in DPBS at 37 °C.

Condition	Solubility (µM)
pH 4	20.3 (4.9)
pH 7.15	33.9 (0.6)
pH 7.15 + 1 mM BS	36.0 (1.1)
pH6.1	21.6 (1.5)
pH 6.1 + 1 mM BS	28.2 (1.1)
Olive oil	1000 (100)

Values expressed as means of three determinations (standard deviation).

**Table 3:** Pyrene fluorescence maximum *F_R_* = *F_III_*/*F_I_* values.

Lipid	*F_R_*
BS micelles ^a^	1.26
FE5:1 micelles	1.17
FE2:1 micelles	1.15
PC liposomes	0.95
LysoPC micelles 0.5 mM	0.86
LysoPC micelles 0.3 mM	0.84

^a^ from [10].

**Table 4:** Quercetin λ_max_ plateau values and % bound to micelles and PC.

pH	Micelles/Lipid	λ_max_ (nm) ^a^	% Bound ^a^
7.15	FE 2:1	376.4 (1.1)	72.7 (2.1)
	FE2:1 + LysoPC ^b^		75.0 (1.5)
	FE 5:1	376.4 (0.7)	52.3 (8.3)
	FE5:1 + LysoPC ^b^		60.0 (1.5)
	FA 2:1	377.1 (0.3)	49.5 (7.1)
	FA 5:1	377.0 (0.4)	25.0 (7.7)
	PC (0.3 mM)	374.8 (0.2)	70.4 (1.7)
	LysoPC (0.3 mM)	374.9 (0.3)	71.7 (1.3)
	BS	377.9 (0.1)	
6.1	PC (0.3 mM)	372.9 (0.2)	82.7 0.9
	LysoPC (0.3 mM)	372.7 (0.1)	76.8 (0.7)
	BS	376.2 (0.1)	

^a^ Mean (SD); ^b^ Half the PC was replaced by LysoPC.
